# Genetics of Retinoblastoma: An Overview and Significance of Genetic Testing in Clinical Practice

**DOI:** 10.3390/genes16091031

**Published:** 2025-08-29

**Authors:** Khaled K. Abu-Amero, Altaf A. Kondkar, Naif A. M. Almontashiri, Abdullah M. Khan, Azza M. Y. Maktabi, Syed Hameed, Saleh AlMesfer

**Affiliations:** 1 Research Department, King Khaled Eye Specialist Hospital and Research Center, Riyadh 11462, Saudi Arabia; nalmontashiri@gmail.com (N.A.M.A.); syhameed@kkesh.med.sa (S.H.); 2 Glaucoma Research Chair, Department of Ophthalmology, College of Medicine, King Saud University, Riyadh 11411, Saudi Arabia; akondkar@gmail.com; 3 College of Applied Medical Sciences and Center for Genetics and Inherited Diseases, Taibah University, Madinah 41477, Saudi Arabia; 4 Pediatric Ophthalmology and Strabismus Division and Ocular Oncology Division, King Khaled Eye Specialist Hospital and Research Center, Riyadh 11462, Saudi Arabia; amkhan@kkesh.med.sa (A.M.K.); smesfer@kkesh.med.sa (S.A.); 5 Department of Pathology and Laboratory Medicine and Ocular Oncology Division, King Khaled Eye Specialist Hospital and Research Center, Riyadh 11462, Saudi Arabia; amaktabi@kkesh.med.sa

**Keywords:** retinoblastoma, clinical genetics, molecular diagnosis, genetic testing, genetic counseling

## Abstract

Retinoblastoma is a rare but malignant pediatric retinal tumor, affecting 1 in 15,000–20,000 live births annually. It arises from biallelic mutations in the *RB1* tumor suppressor gene (chromosome 13q14.2), leading to uncontrolled cell cycle progression. Clinically, it presents as unilateral (60%) or bilateral (40%) disease, with leukocoria and strabismus as hallmark signs. Untreated, retinoblastoma is fatal due to metastatic spread. The disease follows Knudson’s two-hit model: heritable forms (30–40% of cases) involve a germline *RB1* mutation (M1) and a somatic second hit (M2), predisposing to bilateral/multifocal tumors and secondary cancers. Non-heritable cases (60–70%) result from somatic *RB1* mutations or, rarely, *MYCN* amplification (2%). Genetic testing is critical to classify risk (H0, H1, and HX categories), guide surveillance, and inform family counseling. Bilateral cases almost always harbor germline mutations, while 15% of unilateral cases may carry germline/mosaic *RB1* defects. Advanced techniques (Sanger/NGS sequencing for mutation detection, NGS for copy number alterations, and methylation assays) detect *RB1* mutations, CNVs, and epigenetic silencing. Tumor DNA analysis resolves ambiguous cases. H1 patients require intensive ocular and brain MRI surveillance, while H0 cases need no follow-up. Prenatal/preimplantation genetic diagnosis (PGD) can prevent transmission in high-risk families. Emerging research explores additional genes (*BCOR*, *CREBBP*) and *MYCN*-amplified subtypes. Genetic counseling addresses recurrence risks, reproductive options, and long-term cancer monitoring. Integrating genetic insights into clinical practice enhances precision medicine, reducing morbidity and healthcare costs. Future directions include whole-genome sequencing and functional studies to refine therapeutic strategies.

## 1. Introduction

Retinoblastoma is a relatively rare but highly malignant tumor of the retina, affecting one or both eyes of infants and young children. The majority of cases are diagnosed before the age of 5 years, with a median age at diagnosis of 23.2 months [1,2,3,4]. However, a few cases have been documented with an initial diagnosis in individuals up to 21 years of age [5]. It comprises 2.5–4% of all pediatric malignancies [6]. The disease prevalence does vary according to geographic, ethnic, or gender predilections, and the incidence ranges from 1 in 15,000 to 20,000 live births across all populations [1,3,7]. Retinoblastoma has shown a global rise in incidence and prevalence from 1990 to 2021, with the burden falling disproportionately on low- and middle-income countries, particularly in Eastern Sub-Saharan Africa. Although mortality and DALYs have declined overall—thanks to improved therapies and multidisciplinary care in high-income regions—significant disparities remain due to delayed diagnosis and limited access to treatment. The highest burden is seen in female neonates, and projections to 2035 indicate continued increases in disease incidence and disability, despite modest declines in mortality. Socioeconomic factors, genetic predisposition, and sex-based biological differences all contribute to these patterns. Addressing this requires targeted investment in early detection, healthcare access, and regional care infrastructure [8].

Retinoblastoma has a strong genetic component and is often initiated by biallelic mutations in the *RB1* gene, which is located on chromosome 13q14.2 and has 27 exons [9,10,11,12]. *RB1* is a tumor suppressor gene encoding the retinoblastoma protein critical for cell cycle regulation. This protein inhibits the transition from the G1 to S phase of the cell cycle by blocking E2F transcription factors, thereby preventing uncontrolled cell division and retinal tumor formation [12,13,14,15]. Loss of functional RB1 primarily leads to the development of retinoblastoma [10,16]. Retinoblastoma may initiate from RB1-depleted cone precursors in the human retina [14,17,18]. Clinically, retinoblastoma patients commonly present with leukocoria and strabismus, with a mean age at diagnosis of 12 months for bilateral tumors and 24 months for unilateral tumors [19]. Bilateral tumors occur in 40% of retinoblastoma patients, unilateral manifestation is seen in 60%, and trilateral tumors (associated with a midline brain tumor) in about 5% [20,21]. Retinoblastoma has a high survival rate of over 95% in high-income countries, but less than 30% globally [3]. Patients with delayed diagnosis or without treatment face a high mortality rate, with nearly all dying from intracranial extension and disseminated disease within two years of diagnosis [22,23].

Retinoblastoma can be heritable and non-heritable. Based on Knudson’s two-hit hypothesis, two consecutive mutational events are required for retinoblastoma formation [24]. The first hit (M1) is the mutation of the first *RB1* allele, while the second hit (M2) is the loss of the remaining normal allele and duplication of the mutated M1 allele. This biallelic mutation is known as loss of heterozygosity and forms the basis of the genetic subtypes of retinoblastoma [3,6,11,12,25]. The non-heritable retinoblastoma accounts for 60–70% of all retinoblastomas [6], wherein M1 and M2 are somatic mutations occurring in a single retinal cell, usually resulting in unilateral and unifocal tumors with a late onset [11,19]. While 98% of non-heritable retinoblastomas have *RB1* mutations, 2% have somatic amplification of the *MYCN* oncogene without a detectable *RB1* mutation [26]. In contrast, in the heritable form, a germline *RB1* mutation (M1) is present in all body cells with somatic (M2) mutations in multiple retinal cells, increasing the risk of bilateral (80%) and multifocal (67%) retinoblastomas with an early age of onset and secondary non-ocular cancers later in life [6,11,27]. Although less common, heritable retinoblastomas can be unilateral (10–15%), unifocal (33%), or trilateral (5%) [6,11,21,28]. Approximately 75% of germline mutations are sporadic, while the remaining 25% are familial and inherited from an affected parent [6,23,25,29]. Heritable retinoblastoma exhibits autosomal dominant inheritance with high penetrance (over 90%) and high expressivity, accounting for 30–40% of all retinoblastoma cases [6,11,25]. However, low-penetrance and low-expressivity forms have been reported, which may present as unilateral despite being heritable and caused by the same *RB1* gene but different variants [11,30,31,32].

Genetics play a crucial role in many aspects of retinoblastomas, including clinical presentation, choice of treatment modalities, and follow-up for both the child and the family. Genetic testing is ideally indicated in all children diagnosed with retinoblastoma with an unknown *RB1* status. With a high risk of recurrence in future generations, it is also essential for early tumor detection in predisposed children. The heritable form of retinoblastoma demands long-term follow-up for secondary tumors; therefore, genetic testing plays an important role in identifying children who are at risk of developing new tumors. Cutting-edge genomic technologies have greatly improved our ability to detect *RB1* mutations. This has enabled early diagnosis, personalized treatment, and monitoring strategies that can help to reduce the burden of this aggressive tumor. Moreover, this personalized approach will allow clinicians to understand and address the unique needs of each patient, thus enhancing the healthcare quality toward precision medicine.

In this review, we aim to provide clinicians with a simplified and clinically focused genetic testing pipeline. While the other literature may primarily focus on the genetics of retinoblastomas, the role of *RB1* mutations, molecular mechanisms, or technical aspects of mutation detection, this review focuses on the practical application of genetic testing and its importance in the clinical management of retinoblastoma patients, thereby endowing clinicians with the necessary tools and knowledge to optimize patient care.

## 2. Methods

### Search Strategy

A PubMed^®^ search was conducted to identify relevant articles published in the MEDLINE^®^ database. The search terms included combinations of keywords such as “retinoblastoma genetics”, “retinoblastoma mutations”, “*RB1* mutations in retinoblastoma”, “*RB1* mutation analysis”, “genotype-phenotype correlation in retinoblastoma”, “genotype-phenotype correlation in retinoblastoma with *RB1* mutation”, “retinoblastoma genetic testing”, and “genetic counseling in retinoblastoma”. The initial search produced a wide range of articles, which were screened based on their titles and/or abstracts for relevance. The search was limited to studies on human clinical cases and genetic research. Studies addressing the genetics of retinoblastoma in humans, cohort studies, case-control studies, comprehensive reviews, meta-analyses, gene-specific research articles, genetic testing, genotype–phenotype correlations, and genetic counseling were reviewed. Articles without full-text availability, duplication, non-human research, language other than English, preprints, and articles focusing solely on treatment outcomes without genetic data were excluded.

## 3. Diagnosis of Retinoblastoma

The diagnosis of retinoblastoma is clinical and established in a proband by retinal examination with full pupillary dilatation by an ophthalmologist or optometrist. The current clinical diagnostic tools and techniques for early detection of retinoblastoma include fundoscopic evaluation, ultrasonography, fundus fluorescein angiography, optical coherence tomography, and magnetic resonance imaging (MRI) [33,34,35]. These methods help in assessing the characteristic findings associated with retinoblastoma and are essential for making a definitive diagnosis and staging [33,34]. Examination under general anesthesia is strongly recommended for children up to age 3. Histopathological confirmation can be bypassed, as the diagnosis is primarily clinical when the characteristic features are evident [36]. Biopsy is contraindicated due to the risk of tumor dissemination and is rarely performed [3,19]. Newborn eye screening is emphasized in several countries. The red reflex testing screening method is widely used in community settings to identify potential cases of retinoblastoma early [37,38,39]. There is a growing focus on developing digital imaging tools, including mobile apps and the use of artificial intelligence and machine learning to enhance screening capabilities, diagnosis, and classification of retinoblastomas [38,40].

## 4. Heritable Retinoblastoma

Germline pathogenic alterations in the RB1 gene cause heritable retinoblastoma. This form is diagnosed in a proband with retinoblastoma or retinoma who also has a family history of the disease. In the majority of cases, however, there is no known family history. In such cases, genetic testing is needed to identify a heterozygous germline pathogenic or likely pathogenic variant in the RB1 gene to determine if the disease is heritable. According to the RB1 gene mutation database (http://rb1-lsdb.d-lohmann.de) (accessed on 15 June 2025), all pathogenic variants ultimately result in quantitative deficiency or structural disruption of the retinoblastoma protein. A majority of patients with heritable retinoblastoma are heterozygous for RB1 alleles that create a premature termination codon. This results in low quantities or absence of functional RB proteins due to nonsense-mediated mRNA decay, contributing to tumorigenesis [41,42]. It should be noted that, according to the ACMG/AMP variant interpretation guidelines, the terms “pathogenic variant” and “likely pathogenic” are synonymous in a clinical setting, meaning that both are considered diagnostic and can be used for clinical decision-making [43]. In addition, the identification of heterozygous RB1 variants of unknown significance neither establishes nor rules out the diagnosis of retinoblastoma [19]. The key updates and points from the ACMG/AMP guidelines for variant classifications are as follows: (1) Functional evidence must follow a structured four-step process: define disease mechanism, assess assay relevance, validate the assay, and apply results to variants. (2) Variants with >5% frequency in any large continental dataset (≥2000 alleles) can be classified as benign, as per the clarified BA1 criteria. (3) PM2 (absence in population data) has been uniformly downgraded to PM2_Supporting. (4) ACMG is working on subdividing VUS (Variants of Uncertain Significance) by their likelihood of pathogenicity. (5) PS3/BS3 functional evidence uses the same four-step framework as noted above. (6) Gene-disease validity is now guided by ClinGen’s evidence-based gene curation framework. (7) Allele frequency filtering should use the six defined ExAC population subsets. (8) PM2 thresholds should not be the simple inverse of BS1 to avoid misclassification. (9) In silico predictions are encouraged to be concordant, but variability across labs is acknowledged.

## 5. Staging and Genetic Risk Classification

The 8th edition of the American Joint Committee on Cancer (AJCC) staging guidelines for retinoblastoma provides a more accurate way for predicting eye salvage, risk of metastasis, and overall probability of survival. It includes intraocular tumor stages (cT1–cT3), extraocular extension (cT4), regional lymph node involvement (N), and distant metastasis (M). In addition, pathologic staging for enucleated eyes is also included. More importantly, it is the first cancer staging system to integrate heritability (H category) as a formal component of disease classification [44,45]. According to this staging system, retinoblastoma patients, based on their peripheral blood analysis of a germline RB1 pathogenic variant, may be placed in one of the genetic categories H0/H0*, H1, and HX, as shown in Table 1.

## 6. Genetic Testing Approach in Retinoblastoma

Identifying a mutation in the *RB1* gene in the affected patient (proband) is crucial in assessing the genetic risk of heritable retinoblastoma. It allows surveillance of family members who may be at risk of developing retinoblastoma. A stepwise approach is suggested below.

### 6.1. Sample Collection

High-quality DNA can be obtained from peripheral blood or saliva. If the eye has been enucleated, tumor tissue, preferably fresh or flash-frozen, can be used to extract DNA for analysis.

### 6.2. Step 1: RB1 Mutation Analysis

Patients with bilateral, unilateral familial, or unilateral multifocal retinoblastoma should undergo *RB1* gene sequencing using Sanger sequencing or whole-exome sequencing. Peripheral blood is used to identify germline mutations, indicating heritable retinoblastoma. On the other hand, tumor samples are analyzed to detect the second somatic mutation involved in tumorigenesis.

### 6.3. Step 2: Deletion/Duplication Screening

If no mutation is found in Step 1, the next step is to assess for copy number variations (CNVs). Methods like MLPA (multiplex ligation-dependent probe amplification) or array comparative genomic hybridization (aCGH) can be used. MLPA and array CGH are now largely replaced by NGS-based methods for detection of copy number alterations. These methods will allow for the detection of large deletions or duplications in the RB1 gene that are not identified by sequence analysis. The CNV analysis is especially important in children with developmental delays or other congenital anomalies [46,47].

### 6.4. Step 3: Tumor DNA Testing and Additional Analyses

Tumor tissue, if available, can also be tested for *RB1* mutations and deletions/duplications. Ideally, two mutations should be identified in tumor DNA. If pathogenic mutations are found, peripheral blood or saliva DNA should be examined to assess their germline presence.

In the case that no *RB1* mutations are detected in the tumor, a test for *RB1* promoter hypermethylation can be performed to examine epigenetic silencing.

In the case that no methylation is found, test for *MYCN* gene amplification, which accounts for 1–3% of non-heritable unilateral retinoblastoma cases [26,48,49].

### 6.5. Step 4: Loss of Heterozygosity Analysis

If no mutations are found in the above steps, perform an allele loss analysis (loss of heterozygosity) for the *RB1* gene to investigate other mechanisms of gene inactivation.

### 6.6. Step 5: Whole-Genome Sequencing

If all tests are negative, the case may be referred for research-based whole-genome sequencing to explore mutations in other genes potentially involved in retinoblastoma. Such cases must follow appropriate ethical and research protocols, and any novel variants would require functional validation.

## 7. Genetic Risk Assessment

Genetic testing for *RB1* pathogenic variants in the peripheral blood DNA is now a routine part of evaluating retinoblastoma patients. This is particularly important in patients who do not have a family history of the disease. Testing in such patients will allow confirming the diagnosis of H0* non-heritable retinoblastoma and provide better guidance on treatment, follow-up, and counseling.

In bilateral retinoblastoma, a germline *RB1* mutation is found in ~95% of cases using peripheral blood DNA alone [11,28]. If not detected, testing tumor DNA can provide valuable information for recurrence risk assessment. Importantly, since bilateral retinoblastoma is almost always heritable, the risk of passing the mutation to future generations is up to 50%, even when a mutation cannot be confirmed by current testing methods [19].

In unilateral retinoblastoma, tumor DNA should ideally be tested first. If two *RB1* mutations are identified in the tumor tissue alone but neither is found in the peripheral blood or saliva DNA, the case is most likely non-heritable, though mosaicism remains a possibility. In such cases, the proband’s future children may still be at a low but real risk of inheriting the *RB1* mutation [11,19,28]. However, if tumor tissue is unavailable and no mutation is found in the blood, there is still up to a 1.5% chance of an undetected germline mutation [11]. Such children, and potentially their offspring, are recommended to undergo regular eye examinations up to the age of 5 years.

Notably, in patients with unilateral retinoblastoma and no family history, over 15% of cases may still carry a germline or mosaic *RB1* mutation [11,19,25,28]. This can affect their risk of developing new tumors in the future, increase the chance of other cancers, and raise the risk of passing the mutation to their children [50,51].

In case of negative genetic findings where no mutations are identified, further testing for CNV, methylation, and *MYCN* amplification should be pursued (as outlined above). A small percentage (6–8%) of retinoblastoma cases are linked to chromosome 13q14 deletions that are often associated with developmental delays [46,47]. *RB1* promoter hypermethylation, leading to gene silencing, is observed in about 15% of sporadic unilateral, non-heritable cases [52,53,54]. Loss of heterozygosity is observed in 60–70% of tumors from enucleated eyes. Furthermore, the *MYCN* amplification is found in 1–3% of unilateral cases without detectable *RB1* mutations and is considered non-heritable [26,48]. However, it is still unclear if all such *MYCN*-amplified cases are truly non-heritable [55]. Additionally, when all genetic results are inconclusive, clinical presentation and family history can help risk assessment and guide genetic counseling. In general, for RB1-associated retinoblastoma, LOH is essential for tumor initiation, inactivating the second RB1 allele after a germline or somatic first hit. This leads to biallelic loss of RB1, disrupting cell cycle control and enabling tumor development. LOH typically occurs via deletion or mitotic recombination at the RB1 locus. In contrast, MYCN-amplified retinoblastoma usually lacks RB1 mutations entirely. These tumors arise from MYCN oncogene amplification, not the two-hit model. LOH is generally absent, as MYCN amplification alone drives rapid tumorigenesis.

## 8. Current Genetic Testing Methods in Retinoblastoma

For the accurate diagnosis and genetic risk assessment of retinoblastoma, it is essential to have a comprehensive molecular testing strategy to evaluate heritability and family screening. Different methods, complementing each other, are utilized in clinical and research settings to detect pathogenic variants, primarily in the *RB1* gene (Table 2).

Sanger sequencing and next-generation sequencing (NGS) remain the primary methods for detecting single-nucleotide variants and small insertions/deletions that might account for roughly 70–75% of pathogenic *RB1* variants [56,57]. These methods are typically applied to peripheral blood DNA but can also be used on tumor tissue, especially in unilateral cases where tumor-specific mutations may help distinguish somatic from germline events. In the past, MLPA was most widely used to detect large deletions or duplications and detects exon-level copy number changes in an additional 15–20% of retinoblastoma cases [19,28,58]. Recently, it was largely replaced by NGS-based methods for detecting copy number alterations. In patients with syndromic features or developmental delay, aCGH or karyotyping may identify larger chromosomal abnormalities, including deletions involving chromosome 13q14 [46,47]. Additionally, *RB1* promoter hypermethylation testing identifies epigenetic silencing in approximately 10–15% of unilateral, non-heritable cases and can be typically performed on tumor DNA using methylation-specific PCR or genomic sequencing after bisulfite modification of DNA [19,52,53,54,59]. Another supplementary technique is allele-specific PCR (AS-PCR), a method used to identify known mutations even in cases where low-level germline mosaicism is suspected. It is a highly sensitive and efficient method that can detect variant alleles present in as little as 1–5% of sampled cells and confirm family-specific mutation in at-risk relatives [60]. Allele-specific PCR (AS-PCR) was once a reliable tool for detecting known mutations but is now limited by its narrow scope. It cannot identify unexpected variants and lacks the sensitivity needed for low-level mosaicism. NGS offers broader detection, higher sensitivity, and deeper coverage. For familial mutation confirmation, NGS provides a more comprehensive genetic profile. Overall, NGS is now preferred for complex or clinically challenging cases [61]. Moreover, when conventional sequencing is negative but clinical suspicion remains high, RNA-based assays or RNA sequencing (RNA-Seq) can be employed to detect cryptic splice-site mutations or deep intronic variants [62,63]. Currently, however, RNA-seq datasets of retinoblastoma tumors are not published.

In some unilateral cases suspected of a non-heritable, clinically aggressive retinoblastoma subtype, *MYCN* amplification is detected using fluorescent in situ hybridization (FISH) as a standard method, along with MLPA or NGS-based copy number analysis of tumor tissue or cell-free DNA [26,64,65].

In familial cases or when no pathogenic variant is detected despite a strong clinical suspicion, linkage analysis using polymorphic microsatellite markers near RB1 to track the inheritance of the disease allele [66,67,68]. Once a mutation is known in a family, AS-PCR or Sanger sequencing for targeted single-site testing is used for rapid screening of siblings or offspring.

A combination of sequencing, CNV analysis, methylation profiling, and AS-PCR is now the standard care of tumor testing in many genetic diagnostic labs. This approach allows for precise diagnosis and surveillance and guides reproductive and familial risk counseling in retinoblastoma. Newer techniques such as whole-genome sequencing, long-read sequencing, and transcriptome analyses are increasingly being explored in research settings to detect deep intronic changes, regulatory variants, or complex structural rearrangements that are missed by standard testing [55,62,63].

Most routine testing involves screening of the RB1 gene mutation via the NGS method (WES or WGS). Very few laboratories are currently performing other types of testing mentioned above on a routine basis (personal observation).

**Table 2:** Genetic testing methods in retinoblastoma.

Gene (Location)	Technique	Application (Associated Proportion)	Reference Study No.
*RB1* (13q142.2)	DNA sequencing (Sanger or next-generation sequencing)	Detect *RB1* single-nucleotide mutations, small insertions/deletions (70% to 75%)	[56,57]
	Multiplex ligation-dependent probe amplification (MLPA)	Detect large deletions or rearrangements of 1 or several exons in *RB1* (15–20%)	[19,28,58]
	Chromosomal microarray (aCGH) or karyotyping	Detect chromosomal translocations or large gross deletions (6–8%)	[46,47]
	Methylation-specific PCR or sequencing	Detect *RB1* gene silencing of non-heritable retinoblastoma (10–12%)	[52,53,54,59]
	Allele-specific PCR (AS-PCR)	Screen for known mutations in families or at-risk relatives	[60]
	Linkage analysis	Detect mutant gene in families with 2 or more affected relatives when mutation(s) cannot be detected with conventional techniques.	[66,67,68]
*MYCN* (2p24.3)	Fluorescent in situ hybridization (FISH), MLPA, or NGS-based copy number analysis	Detect *MYCN* amplification on 2p on tumor tissue (1–3%)	[26,48,55]
Genome-wide	Whole-genome sequencing	Screen *RB1*/*MYCN*-negative retinoblastoma	[55]
	RNA-Seq/Transcriptomics	Detect non-coding RNA changes, alternate splicing events, and novel RNAs	[62,63]

## 9. Importance of Genetic Testing

Genetic testing is critical to classify retinoblastoma as either sporadic or inherited. This classification enables prognosis, helps risk assessment, and guides surveillance strategies. Thus, genetic testing has a central role in the diagnosis, management, and counseling of patients with retinoblastoma and their families, as summarized in Table 3. While bilateral retinoblastoma patients almost always harbor a germline *RB1* pathogenic mutation, notably, approximately 15% of unilateral cases, including those with no family history, are also associated with germline or mosaic *RB1* pathogenic variants [11,19,25,28]. Identifying such mutations helps clinicians assess the prognosis, including the risk of new tumors in one or both eyes, the likelihood of developing brain tumors (e.g., pinealoblastoma), and other non-ocular cancers later in life [19,69]. Genetic testing also provides targeted surveillance by helping decide which family members may need testing or follow-up. As such, genetic testing should be recommended to all patients with retinoblastoma, especially when the *RB1* status is unknown, irrespective of tumor laterality, age at diagnosis, or family history [50,51].

Beyond helping to understand a patient-specific risk assessment, genetic testing also offers the benefits of early-stage recognition, monitoring, and timely intervention of at-risk relatives, especially siblings and children. It helps parents and adult survivors make informed decisions on reproductive planning and supports genetic counseling. Additionally, it prevents unnecessary tests and follow-ups, reducing stress and medical costs for family members who do not carry the RB1 mutation (H0).

For H0* patients (those with no family history), identifying the mutation can confirm whether the retinoblastoma is sporadic or hereditary. This distinction is important for guiding future surveillance and understanding long-term cancer risks. Furthermore, in families with a known *RB1* mutation, prenatal or preimplantation genetic testing and in vitro fertilization (IVF) can be considered. This allows for planning early delivery and monitoring of H1 infants for very small tumors using advanced imaging like optical coherence tomography and initiates precise early intervention, such as laser therapy, before tumors become clinically visible [70,71].

## 10. Genotype–Phenotype Correlations

In the majority of families with heritable retinoblastoma, all members who inherited the germline pathogenic variant develop multiple tumors in both eyes, presenting as bilateral multifocal disease. It is not unusual to find, however, that the founder (i.e., the first person in the family to have retinoblastoma) has only unilateral retinoblastoma. These families may have *RB1* null alleles that are altered by frameshift or nonsense variants, or the founder may be mosaic for the pathogenic *RB1* allele. With a few exceptions, *RB1* null alleles show complete penetrance (>99%) [72,73,74]. In such families, almost everyone who inherits the mutation eventually develops retinoblastoma.

A small proportion of families (less than 10%) show a “low-penetrance” phenotype with reduced expressivity (i.e., increased prevalence of unilateral retinoblastoma) and reduced penetrance (i.e., ≤25%) [30,72,75]. This low-penetrance phenotype is usually associated with *RB1* in-frame, missense, or distinct splice site variants, certain indel variants in exon 1, or pathogenic variants in the promoter region. These mutations may allow for partial function of the RB1 protein, reducing the severity or likelihood of tumor development. In these families, the risk to relatives and offspring may be lower but still requires careful evaluation [75].

A third category of families exhibits a parent-of-origin effect. In such cases, the clinical outcome shows differential penetrance and expressivity depending on whether the *RB1* mutation was inherited from the mother or the father. This phenomenon is attributed to known imprinting effects or modifying genes in affected individuals [76,77,78,79].

In addition, cytogenetically visible deletions of 13q14 that include genes adjacent to *RB1* may cause developmental delay and mild-to-moderate facial dysmorphism, a condition known as 13q deletion syndrome [47]. These large deletions of 13q14 show reduced expressivity, and individuals may have unilateral retinoblastoma or absence of retinoblastoma [46]. Contiguous loss of neighboring genes such as *MED4*, which is located centromeric to *RB1*, may modulate *RB1* expression or tumor susceptibility [80].

Another distinct clinical entity is *MYCN*-amplified retinoblastoma, which occurs in a few percent of unilateral, non-heritable cases [26]. These tumors are driven by high-level amplification of the *MYCN* oncogene rather than *RB1* mutations and typically present in infants younger than 12 months. Such tumors are often aggressive, fast-growing, and not associated with a germline mutation, i.e., they do not carry heritable risk for the child or family. However, they require early recognition due to their aggressive clinical course [19,26].

Mosaicism is another important factor in genotype–phenotype correlation. Individuals with mosaic *RB1* mutations may have a milder phenotype, such as unilateral disease or fewer tumors, and standard blood testing may fail to detect the pathogenic variant. In such cases, testing tumor tissue and using sensitive methods (e.g., allele-specific PCR) can help confirm the diagnosis [60].

Understanding the genotype–phenotype patterns is critical not only for predicting clinical outcomes but also for tailoring surveillance, counseling families, and deciding on reproductive options. As new data continue to emerge, especially with the use of whole-genome sequencing and epigenetic profiling, our ability to predict risk and personalize care will continue to improve.

## 11. Surveillance and Follow-Up Strategy Based on RB1 Gene Status

The frequency and intensity of surveillance in patients and relatives with *RB1* alterations should be guided by their clinical presentation and *RB1* genetic status [11,19,28,45,72,81,82,83,84]. A precise classification of H1, H0, H0*, or HX helps guide how often and how long children and at-risk relatives need to be monitored, as outlined in Table 4.

H1 retinoblastoma patients are at high risk of developing new and bilateral tumors, or trilateral disease in some cases, especially in the first five years of life. They should undergo eye examinations under general anesthesia every 2–4 weeks during the first year of life, followed by every 1–2 months until age 2, then every 3–6 months until age 5. Though some may suggest performing brain MRI every 6 months until age 3–5 for early detection of trilateral retinoblastoma, this is not yet supported by strong evidence, and some authors recommended against that, as the overall survival from TRB was not improved as a result of screening, and many false-positive results required additional, subsequent MRI scans with anesthesia [85,86]. Later, they may need annual surveillance for second primary malignancies. Genetic counseling and testing for siblings and offspring are essential, as the inheritance risk is 50% [72,81,82,84,87].

In addition, patients with retinoma, a benign, non-progressive retinal lesion, who carry germline *RB1* mutations (H1) with no full-blown retinoblastoma, still carry a risk for second non-ocular malignancies. Such patients, and those who present with non-ocular primary malignancies linked to *RB1* mutations (e.g., sarcomas, melanomas, or pinealoblastomas), should undergo long-term surveillance for secondary cancers and receive genetic counseling [19,69].

For patients classified as H0, there is no need for continued ocular surveillance beyond initial treatment, and relatives do not require genetic testing or follow-up [69]. However, H0* cases carry a small risk of undetected mosaicism. These individuals should be monitored every 3 months until age 3 and then every 6 months until age 5 [19]. Family members may not require testing unless new clinical signs or a family history emerge [26,72,83].

Lastly, in HX cases, follow-up should be guided by clinical presentation. For bilateral cases or early-onset unilateral disease, the patient should be managed like H1 until genetic status is clarified. EUA frequency should match H1 guidelines until testing results are available. Genetic counseling is strongly recommended [81,82].

## 12. Other Potential Genes Associated with Retinoblastoma

While RB1 remains the primary gene associated with retinoblastoma, and *MYCN* amplification accounts for a small subset of non-hereditary, unilateral cases, emerging research has identified a number of additional genes that may play a role in retinoblastoma development or progression. Genes such as *BCOR*, *CREBBP*, *ATRX*, *SMARCB1*, and *ARID1A*, which are involved in chromatin remodeling, transcriptional regulation, or DNA repair, have been found to be mutated or dysregulated in some retinoblastoma tumors [49,88,89,90,91]. In addition, the *CRB1*, *NEK7*, *SOX4*, and *NUP205* genes, identified through the genome-wide multi-omics approach in RB1-deficient tumors, have emerged as novel candidate genes associated with retinoblastoma tumorigenesis [49,92]. Furthermore, *MDM4*, *KIF14*, *DEK, E2F3*, and *CDH11* are consistently identified candidate oncogenes and tumor suppressor (*CDH11*) genes in retinoblastoma that are associated with recurrent chromosomal gains at 1q, 6p, and 13q and linked to tumor progression, genomic instability, and therapeutic resistance [93,94,95,96]. *PCDH8*, a member of the cadherin superfamily, is also an emerging tumor suppressor gene in retinoblastoma and under early investigation [47]. Moreover, alterations in genes related to the p53 pathway and cell cycle control, such as *TP53* and *MDM4*, have also been observed in a subset of aggressive or treatment-resistant cases [92]. Some of these may act as cooperating mutations in *RB1*-deficient tumors or influence tumor progression, mostly in cases with chromosomal instability. Likewise, several genetic polymorphisms in multiple genes have also been linked to retinoblastoma susceptibility [97,98,99]. However, the direct role of none of these genes in retinoblastoma is yet fully understood and warrants further investigation for their potential diagnostic or prognostic relevance; nonetheless, they highlight the expanding genetic landscape of retinoblastoma beyond *RB1* and *MYCN*. Further studies using whole-exome/genome sequencing, epigenetic profiling, and functional validation are essential to determine the clinical significance of these genes and their utility in diagnosis, risk stratification, or as therapeutic targets. The RB1 gene is the central driver of retinoblastoma and the most established gene. Biallelic inactivation of RB1, either germline or somatic, is necessary for tumor development, especially in heritable and bilateral cases. MYCN amplification accounts for a small subset of aggressive, unilateral, non-heritable tumors lacking RB1 mutations and is the second most relevant alteration. TP53 is not directly implicated in retinoblastoma initiation but may play a role in secondary malignancies, especially post-radiation in RB1-mutant patients. Genes like MDM2 and MDM4 can influence tumor behavior by regulating p53 but are not causative. Additional alterations such as BCOR and CREBBP have been observed in tumor genomes, though their roles are secondary. Genetic testing always begins with RB1, with MYCN considered when RB1 is intact. Other genes currently have limited diagnostic or therapeutic value. Overall, RB1 dominates the genetic landscape, with MYCN as a critical but much rarer exception.

## 13. Genetic Counseling in Retinoblastoma

Genetic counseling for retinoblastoma poses distinct problems owing to the intricacies of genetic and clinical characteristics. Although retinoblastoma is predominantly a monogenic condition associated with the *RB1* gene, differences in laterality (unilateral versus bilateral) markedly affect the occurrence of harmful mutations. Bilateral instances consistently exhibit a germline *RB1* variation, whereas unilateral cases have a 15–20% probability. Mosaicism and imperfect penetrance hinder the evaluation of family history and the estimation of recurrence risk.

### The Primary Aspects of Genetic Counseling for Retinoblastoma Are as Follows:

- The “patient” encompasses not just the affected individual but also parents, siblings, and extended family members. Genetic counseling is essential for both affected children and adult survivors at risk of secondary malignancies.
- Counseling Referrals: Patients with unilateral retinoblastoma, particularly those lacking a familial history, frequently receive insufficient counseling concerning the possibility of germline variants. Genetic testing is crucial for both bilateral and unilateral instances to guide treatment and management methods.
- Role of Genetic Counselor: Counselors evaluate risk, provide intricate information, and develop a tailored care plan. Effective counseling necessitates an awareness of retinoblastoma and collaboration within a multidisciplinary team.
- Preparing Families: Early introduction of families to the multidisciplinary team helps alleviate anxiety and enhance comprehension of the care process. Documenting familial lineage in a pedigree is essential for comprehending genetic risks.
- Confounding Factors: Conditions such as chromosome 13q14 deletions and mosaicism may confuse genetic evaluations. Low-penetrance mutations and changing familial histories require continuous risk reassessment.
- Prenatal Diagnosis: Families with identified *RB1* variants should contemplate options such as prenatal diagnosis and preimplantation genetic diagnosis (PGD) for reproductive planning.
- Technological Limitations: Genetic testing has constraints, including the possibility of false negatives and undetected variants, which must be explicitly conveyed to families. Ultimately, proficient genetic counseling for retinoblastoma is essential for informed decision-making concerning therapy, recurrence risks, and family planning. Incorporating genetic knowledge into clinical practice can markedly enhance the care and support offered to impacted families. Progress in tumor DNA analysis and the comprehension of RB genetics will improve patient care and counseling methodologies.

## 14. Preimplantation Genetic Diagnosis to Prevent the Transmission of Pathogenic RB1 Variants in Retinoblastoma

Preimplantation genetic diagnosis (PGD) is a promising technique to eradicate retinoblastoma, since it allows couples with a hereditary risk to pick unaffected embryos during in vitro fertilization. By screening embryos for the *RB1* gene mutation linked to retinoblastoma, parents can prevent passing on the genetic propensity to their children. This targeted selection greatly reduces the disease’s prevalence within families. As more families use PGD, the prevalence of retinoblastoma may decrease over generations, perhaps leading to the condition’s extinction. In addition, PGD reduces the emotional and financial difficulties associated with illness management. A case study in 2007 reported the first successful use of PGD for retinoblastoma in the UK and highlighted the feasibility of PGD in retinoblastoma [100]. In a more recent comprehensive study, the economic advantages of PGD in managing heritable retinoblastoma showed great benefits in reducing the cost of managing retinoblastoma patients. The study also indicates that subsidizing PGD could improve access and reduce socioeconomic inequalities in reproductive choices for families at risk and substantially improve the quality of life [101].

## 15. Conclusion and Closing Remarks

### The Following Are the Concluding Remarks from This Review:

- Genetic testing is pivotal in classifying retinoblastoma as heritable or sporadic, guiding prognosis, surveillance, and family counseling.
- It enables early diagnosis, personalized treatment, and monitoring, significantly improving patient outcomes.
- Bilateral retinoblastoma cases almost always harbor germline *RB1* mutations, while ~15% of unilateral cases may also carry germline or mosaic mutations.
- Identifying *RB1* mutations helps assess risks of new tumors, trilateral retinoblastoma, and secondary cancers.
- Surveillance strategies must be tailored based on *RB1* status (H1, H0, H0*, and HX) to optimize follow-up frequency and interventions.
- Genetic counseling is essential for families, addressing reproductive planning and risk assessment for relatives.
- Prenatal and preimplantation genetic diagnosis (PGD) offer opportunities to prevent disease transmission in high-risk families.
- Advances in genomic technologies, such as whole-genome sequencing, enhance mutation detection and understanding of genotype–phenotype correlations.
- Low-penetrance *RB1* variants and mosaicism complicate risk predictions, necessitating sensitive testing methods.
- MYCN-amplified retinoblastoma, though rare, requires early recognition due to its aggressive nature.
- Emerging genes beyond *RB1* (e.g., *BCOR*, *CREBBP*) highlight the expanding genetic landscape of retinoblastoma.
- Multidisciplinary collaboration is crucial for integrating genetic insights into clinical practice.
- Precision medicine approaches, informed by genetic testing, reduce unnecessary interventions and healthcare costs.
- Continued research is needed to elucidate the roles of novel genes and epigenetic modifications in retinoblastoma.
- Ultimately, genetic testing empowers clinicians and families to make informed decisions, improving care and quality of life for retinoblastoma patients.

## Figures and Tables

**Table 1:** *RB1* genetic risk of heritable retinoblastoma.

Category	*RB1* Gene Status	Reference Study No.
H0	Patients who did not inherit a known familial germline *RB1* pathogenic variant confirmed by molecular genetic testing and have normal *RB1* alleles in blood, tested with demonstrated high-sensitivity molecular genetic assays	[19,44,45]
H0*	Is assigned in patients with unilateral retinoblastoma or retinoma with no germline *RB1* pathogenic variant identified on molecular genetic testing but have <1% residual risk of an *RB1* pathogenic variant due to undetectable mosaicism	
H1	Presence of bilateral retinoblastoma, trilateral retinoblastoma (retinoblastoma with intracranial central nervous system midline embryonic tumor), a patient with a family history of retinoblastoma, or molecular identification of a germline *RB1* pathogenic mutation.	
HX	Patients with unknown or insufficient evidence of a germline *RB1* pathogenic variant	

**Table 3:** Importance of genetic testing in retinoblastoma.

Why Test?	Key Benefits
Identify heritable cases	~100% of bilateral and ~15% of unilateral cases have germline *RB1* mutations (H1), even without family history
Guide prognosis	Germline *RB1* mutations are linked to the risk of new tumors (both eyes), trilateral retinoblastoma, and secondary non-ocular cancers
Support targeted surveillance	Helps tailor follow-up schedules for patients and at-risk relatives
Enable family counseling	Informs reproductive decisions, including prenatal or preimplantation genetic testing
Prevent unnecessary testing	*RB1*-negative (H0) relatives avoid unnecessary exams, anesthesia, and imaging
Allow early intervention	Prenatal H1 diagnosis enables early delivery and optical coherence tomography-guided detection of subclinical tumors for timely laser therapy
Reduce costs	Improves care efficiency and reduces long-term screening and treatment costs

**Table 4:** Surveillance and follow-up in at-risk retinoblastoma patients as per *RB1* status.

*RB1* Status (Clinical Scenario)	Definition	Surveillance for Patient	Clinical Evaluation Frequency	Family Follow-Up	Genetic Counseling	Reference Study No.
H1 (Retinoblastoma)	RB with germline *RB1* mutation	EUA and long-term cancer surveillance	Birth–1 yr: every 2–4 weeks; 1–2 yrs: every 1–2 months; 2–5 yrs: every 3–6 months; annual exams thereafter. Brain MRI every 6 months until age 5 for trilateral RB	Predictive testing for siblings/offspring; follow EUA if positive	Strongly recommended; 50% risk of transmission	[11,19,28,45,72,81,82,83,84]
H1 (Retinoma)	Retinoma (no active RB) with germline *RB1* mutation	No ocular treatment needed; long-term surveillance for second cancers	Annual physical exam; oncology review as per age and treatment history	Genetic testing for family members; EUA for at-risk children up to age 5	Strongly recommended; same transmission risk as H1	
H1 (Non-ocular tumor)	*RB1* mutation carrier with non-ocular malignancy	Surveillance for second tumors; lifestyle and cancer prevention counseling	Annual follow-up; MRI or targeted screening if high-risk tumor type	Genetic counseling and *RB1* testing in first-degree relatives	Strongly recommended, especially if no ocular RB history	
H0	No *RB1* mutation in blood/tumor; confirmed somatic	No ocular or systemic follow-up needed post-treatment	None (unless clinically indicated)	No family testing or screening required	Not needed; no risk to offspring	
H0*	No mutation in blood; tumor not tested	Surveillance to rule out mosaicism	Every 3 months until age 3; every 6 months until age 5	Case-by-case counseling; test relatives only if signs/symptoms emerge	Generally low risk; tailored counseling is advised	
HX	Genetic testing is incomplete or unavailable	Follow clinical risk; manage as H1 if high risk	Same as H1 until testing is completed	Testing and counseling should be offered	Strongly recommended; risk unclear; testing advised before pregnancy planning	

RB: retinoblastoma; EUA: examination under general anesthesia; MRI: magnetic resonance imaging.

## Data Availability

No new data were created or analyzed in this study. Data sharing is not applicable to this article.
